# Driving behaviour in adults with attention deficit/hyperactivity disorder

**DOI:** 10.1186/s12888-015-0566-y

**Published:** 2015-07-28

**Authors:** Madeleine J. Groom, Editha van Loon, David Daley, Peter Chapman, Chris Hollis

**Affiliations:** Division of Psychiatry and Applied Psychology, University of Nottingham, Institute of Mental Health, University Of Nottingham Innovation Park, Triumph Road, Nottingham, NG7 2TU UK; School of Psychology, University Of Nottingham, University Park, Nottingham, NG7 2RD UK; Department of Child and Adolescent Psychiatry, Nottinghamshire Healthcare NHS Trust, E Floor, South Block, Queens Medical Centre, Derby Road, Nottingham, NG7 2UH UK

**Keywords:** ADHD, Driving, Cognition, Road safety, Hyperactivity-impulsivity, Inattention

## Abstract

**Background:**

Little is known about the impact of cognitive impairments on driving in adults with ADHD. The present study compared the performance of adults with and without ADHD in a driving simulator on two different routes: an urban route which we hypothesised would exacerbate weak impulse control in ADHD and a motorway route, to challenge deficits in sustained attention.

**Methods:**

Adults with (*n* = 22, 16 males) and without (*n* = 21, 18 males) ADHD completed a simulated driving session while eye movement data were recorded simultaneously. Participants also completed the Manchester Driving Behaviour Questionnaire (DBQ) and the Conners Adult ADHD Rating Scale (CAARS). Measures of driving performance included average speed, proportion distance travelled over speed limit (speeding) and lane deviation. These variables and the eye movement measures (spread of fixations, mean fixation duration) were compared between groups and routes. Also, driving behaviours, including responses to programmed events, were categorised and the frequencies within categories were compared between groups. Finally, speech analysis was performed to compare emotional verbal expressions during driving between groups.

**Results:**

ADHD participants reported significantly more Violations and Lapses on the DBQ than control participants and significantly more accidents. Average speed and speeding were also higher but did not interact with route type. ADHD participants showed poorer vehicle control, greater levels of frustration with other road users (including greater frequencies of negative comments) and a trend for less safe driving when changing lanes/overtaking on the motorway. These effects were predicted by hyperactive/impulsive CAARS scores. They were also more likely to cause a crash/near miss when an event occurred on the urban route.

**Conclusions:**

The results suggest that difficulty regulating and controlling impulsive behavior, reflected in speeding, frustration with other road users, less safety when changing lanes on the motorway and a greater likelihood of an accident following an unexpected event, underlie impaired driving in ADHD. Hyperactivity/impulsivity symptoms correlated with these indices. Deficits in sustained attention seemed to play a lesser role in this particular study, although further research is needed to determine whether effects on attention emerge over longer periods of time and/or are influenced by the novelty of the simulator environment.

## Background

Attention deficit hyperactivity disorder (ADHD) commonly persists into adulthood with prevalence in adult community samples recently estimated at 2.5 % [1]. The cognitive impairments associated with ADHD in childhood continue into adulthood [2] but relatively little is known about the impact of these difficulties on activities of daily living in adulthood such as driving.

Significantly increased rates of driving accidents (including those for which the driver was liable) and driving prosecutions have been reported in adults diagnosed with ADHD (reviewed in [3–5]). These adults are more likely to exceed the speed limit, have poorer vehicle control [6], express frustration and anger with other road users [7, 8] and be distracted when driving [9] than adults without ADHD and experience greater deterioration in driving performance when under the influence of alcohol [10, 11]. Previous research investigating cognitive deficits in ADHD suggests a number of processes that may be responsible for poorer driving in this population. For instance, weak inhibitory motor control [12], poor monitoring and evaluation of performance [13], reduced arousal and attentional lapses [14, 15], associated with dysfunction in dopaminergic and noradrenergic brain systems [16, 17], may each exert some influence. In healthy adults, self-reported deficits in the regulation of attention and impulse control predict driving errors (e.g. failing to check mirrors before changing lanes, failing to notice pedestrians crossing) and violations (e.g. driving too close to the car in front, disregarding the speed limit) respectively [18]. Inattention and impulsivity are cardinal features of ADHD, and may be related to different causal pathways and cognitive mechanisms [19]. It seems likely therefore that these factors will also prove important during driving in individuals with ADHD and also that they might predict different aspects of driving performance.

It is known that cognitive impairments in ADHD are reduced by contextual factors that increase motivation [20] and worsen during long, boring cognitive tasks with low incentives [12, 14], thought to arise from a failure to regulate arousal and motivational state [15]. Contextual factors such as the type of road and surrounding environment are known to influence driving in healthy adults [21] and may also influence driving performance in ADHD. Specifically, motorway driving might lead to more driving errors in ADHD (lane deviations, failing to notice signs/hazards, slow reactions to events) due to difficulties sustaining attention in low stimulation environments, as shown previously [22] whereas urban routes might lead to more driving violations (exceeding the speed limit, expressing verbal frustration with other drivers) due to exacerbation of poor cognitive/emotional impulse control in a stimulating environment.

The present study compared the performance of adults with and without ADHD in a driving simulator in which two different routes were presented: urban and motorway. We chose to use a driving simulator as this enhances the realism of the driving experience compared with studies using a standard computer assessment. Each driving route contained specific events occurring at pre-determined times. Performance indicators including speed, proportion of distance over the speed limit and lane deviation were compared between groups and routes. Considering the influence of motivational incentives on parameters of attention and impulse control in ADHD [12, 14, 23] we decided not to provide performance-based monetary incentives in case these reduce the impact of impairments in attention and impulse control on driving performance, which may have influenced the findings of previous studies [22, 24]. Instead, a fixed inconvenience allowance was given to all participants after they completed all study assessments. The groups were also compared on reactions to timed events, behavioural and verbal expressions of frustration/anger with other road users and general driving performance. In particular, we examined emotional speech during the simulated drive based on evidence that ADHD is associated with emotional dysregulation [25] and that this can be exacerbated in reaction to events that are outside of direct control [26]. We reasoned that events within the driving scenario, including the actions of other road users, could further exacerbate this tendency in the ADHD group, manifesting in greater use of negative emotion words, including swearing.

As well as standard measures of driving performance, eye movements provide an index of the allocation of visual attention with millisecond precision and tend to be atypical in ADHD [27]. In driving research, a widely used measure of the allocation of visual attention is the horizontal and vertical spread of search, which varies with road type and danger level [28], driving experience [29, 30] and mental workload [31]. Only one previous study reported measuring eye movements in ADHD, finding no differences between adults with ADHD and controls [32]. However, the sample sizes were small (5 ADHD, 5 controls) and ADHD diagnosis was not well established. In addition, participants were not tested in a driving simulator and so the lack of realism may have influenced the findings. The present study measured eye movements during a carefully controlled simulated driving experiment using a larger, well-defined clinical ADHD sample. We predicted significantly reduced gaze concentration (greater spread of vertical and horizontal eye movements) and fixation duration in the ADHD group compared with controls, particularly on the motorway route, where the allocation of visual attention is expected to be adversely affected by difficulty sustaining attention.

## Methods

### Participants

Sixty-four participants aged 18 to 54 years with a UK driving licence were recruited to this study. Participants in the ADHD group (*N* = 29, 12 females) were patients undergoing assessment or receiving care from the Adult ADHD Clinic, Nottinghamshire Healthcare NHS Trust, U.K. Of 74 potential participants initially contacted about the study, 21 did not drive, 18 were not interested or did not feel able to take part, 1 was outside the age criteria (18 to 55 years) and 5 withdrew after initially agreeing to take part, providing a sample of 29. Participants in the ADHD group had a confirmed lifetime and current diagnosis of ADHD according to DSM-IV-TR criteria. All were recruited through an adult ADHD service led by a Consultant Child & Adolescent Psychiatrist (CH) with extensive experience of assessment and diagnosis of ADHD across the lifespan. Diagnosis was made as part of thorough clinical assessment prior to referring patients for participation in the study. Current and lifetime ADHD diagnosis was established by conducting the Diagnostic Interview for ADHD in Adults (DIVA 2.0 [33]), childhood developmental history and comprehensive psychiatric assessment.Scores were also obtained from the Conners Adult ADHD Rating Scales (CAARS self- and observer-report) [34] and Autism Quotient (AQ) [35]. All participants met current and lifetime criteria for ADHD diagnosis.

Of the 29 participants with ADHD, 17 were taking stimulant medication, two were taking non-stimulant medication (atomoxetine), one was taking bupropion and nine were not taking any medication. The study was approved by the Nottingham Research Ethics Committee and by the Research & Development department of Nottinghamshire Healthcare Trust. All participants provided fully informed consent.

Control participants (*N* = 35, 11 females) were recruited through posters displayed on the University of Nottingham campus, U.K. and in local community centres. Volunteers were eligible to take part if they were aged 18 to 55 years, held a driving licence and had never received a diagnosis of ADHD or autism spectrum disorder (ASD).

All participants completed the CAARS (self-report) [34] to assess presence and severity of ADHD symptoms and the AQ [35] to measure signs of ASD. These scales are well-established tools for screening for ADHD and ASD symptoms, demonstrating good test-retest reliability (correlation .89 for CAARS and .7 for AQ) and moderate to excellent internal consistency (Cronbach’s alpha .86 to .92 for CAARS and .63 to 77 for AQ) [35, 36]. Of 29 participants in the ADHD group, 6 met/exceeded the threshold for risk of ASD. These participants were not excluded from the study due to the high rates of ASD symptoms in the adult ADHD population. Instead, the possible influence of ASD symptoms on the dependent variables of interest was examined during statistical analysis. Five potential control participants were excluded from the study as their score on the CAARS self-report exceeded the screening cut-off point for ADHD (score >65 on the CAARS ADHD Index). Of the remaining 30 control participants, none met the threshold for ASD (score >32 on the AQ).

### Apparatus and stimuli

#### Driving simulator and Eye-tracking apparatus

The driving simulator used was the Nottingham Integrated Transport and Environment Simulation facility’s high fidelity system (NITES 1). This driving simulator consists of a fully instrumented BMW Mini housed within a 360° projection dome mounted on a Bosch Rexroth six degrees-of-freedom motion platform. The wing mirrors of the vehicle contain LCD screens with a graphical representation of the rear view and a sound system provides realistic vehicle and traffic noise.

The driving scenario consisted of three different road types. The scenario started in a built-up urban area, which required constant shifting of attention and monitoring and evaluation of performance. After driving 2.1 miles in the urban area, participants reached a single carriageway, which they drove on for 4.6 miles until they reached a three lane motorway which was followed for 9.8 miles. The motorway section contained little traffic, therefore providing a monotonous and low stimulation environment. The single carriageway section was included only to improve the realism of driving from the urban area onto a motorway. All three parts of the drive contained short sections with a speed limit that was lower than the default speed limit for that road type (i.e. 20 mph in the urban area and 40 mph on the carriageway and the motorway). Along the route, five events were programmed to occur, three in the urban area and two on the motorway. Examples of these are pedestrians stepping onto the road and a car suddenly pulling out in the urban area and sudden slowing down of traffic due to an accident on the motorway. Continuous driving performance measures obtained from the simulator were average speed, the proportion of the distance travelled in excess of the speed limit (in excess of 10 % of the speed limit for that road section plus two miles), the coefficient of variation of velocity and the standard deviation of lateral position.

Eye movements were recorded simultaneously by two Seeing Machines FaceLAB 5 eye tracking systems using four cameras. Measures obtained were mean fixation duration and the standard deviation of gaze coordinates (spread of search) for both the horizontal and vertical axes.

#### Self-report measures of driving performance

The Manchester Driving Behaviour Questionnaire (DBQ) [37], one of the most widely used questionnaires in driving research, was used to assess self-reported driving. It consists of items measuring three different components: errors, violations and lapses. Errors reflect mistakes due to misjudgements and failures of observation such as failing to check mirrors before changing lanes or attempting to overtake someone that you hadn’t noticed to be signalling a right turn. Violations are deliberate deviations from what is considered to be safe driving (illegal or not) such as speeding, tailgating, undertaking and jumping a red light. Lapses refer to less serious failures of attention or memory such as taking the wrong exit on a roundabout after misreading the signs or having no recollection of the road you have just been travelling or where you parked your car. Violations and errors are significant predictors of self-reported accidents [38].

#### Observational measures of driving performance

To obtain a more detailed indication of participants’ driving behaviour, a coding scheme consisting of 23 items in seven categories was developed to score driving errors and violations (see Table 3). One independent observer naïve to the purpose of the study and blinded to group allocation coded all 43 videos showing a reconstruction of the participant’s drive with their eye movements overlaid as well as a video (including audio) of the participant. Additionally, responses to events in the urban section were categorised as ‘crash or near miss’, ‘appropriate response’ (slowing down or changing lane) or ‘missed hazard due to fast driving’. Responses to events on the motorway were coded as ‘did not slow down’, ‘slowed but not below speed limit’ or ‘slowed below speed limit’. A second observer coded 55 % of the urban and motorway driving scenarios. Inter–reliability scores were calculated and Kappa ranged from 0.70 (observed behaviour in the seven categories) to 0.87 (responses to events). Only the data of the first observer are reported. Finally, the start and duration of ocular fixations following an event were coded, however the inter-rater reliability was poor and so the data were not entered into statistical analysis.

To gain an additional measure of driving behaviour spontaneous comments made during the drive were transcribed and processed by the Linguistic Inquiry and Word Count (LIWC) text analysis software [39]. This software calculates the degree to which different categories of words occur based on a dictionary of around 4500 words and word stems with 82 language dimensions. Previous research has shown excellent levels of internal consistency (e.g. Cronbach’s alpha = .97 for emotion words) (measured as the consistency between words within a category) of this software and acceptable external validity [39]. The categories used in the present study were positive emotion, negative emotion, anger, swearing and anxiety. The frequency of each category of words was recorded for each participant (words can be counted in more than one category). In addition we computed the total number of comments made and the number of words per comment.

### Procedure

All participants in the ADHD group who were on stimulant medication withdrew from medication for 24–36 h (depending on their medication regime) before the study simulator session. Before taking part in the study, participants were asked to fill in an informed consent sheet, a driving and health questionnaire, the CAARS (self-report), AQ and DBQ. After filling out the forms, participants were seated in the simulator and were given instructions about its use and safety procedures. Following calibration of the eye trackers a 5-min practice drive was completed with the experimenter present in the dome. Automated verbal directions were given throughout the route. After completion of this drive, participants completed the 16-item Kennedy Simulator Sickness checklist Questionnaire (SSQ) [40] to ensure they were not experiencing too many symptoms of simulator sickness at this point. In case of a high score, participants were withdrawn from the remainder of the study. The experimenter then left the dome and after a short break during which the motion platform was activated, the participants drove the experimental part of the route, which took about 25 min. After completing the experimental drive, participants were asked to fill in the simulator sickness checklist again as well as a post-trial consent form to ensure that any feelings of discomfort they may have experienced had subsided. Participants received an inconvenience allowance for taking part.

### Data analysis

#### Self-report measures of driving behaviour and driving history

Scores on each factor of the DBQ (errors, lapses, violations) and items assessing driving history were compared between ADHD and control groups using independent-samples t-tests.

#### Simulator driving performance and eye movement measures

The continuous driving performance measures (average speed, proportion of distance travelled in excess of speed limit, coefficient of variation of velocity and standard deviation of lateral position) and the eye movement measures (mean fixation duration, standard deviation of gaze coordinates (spread of search) for both the horizontal and vertical axes) were each entered into separate mixed design ANOVAs. Each ANOVA was designed to assess the between-subject effect of Group (Control, ADHD) and the within-subject effect of Road Type (Urban, Motorway) on these variables, and the Group*Road Type interaction.

#### Observational coding of driving behaviours

##### Driving performance

The categories identified from the observational coding of driving behaviours were compared between groups by first computing the total frequency of each behaviour within each category and then comparing the group mean frequencies using multivariate ANOVA across items within each category.

##### Responses to events

To compare the type of response to urban and motorway events between groups, the chi-square statistic was computed to determine whether there were group differences in allocation of participants to categories.

##### Emotional speech

The frequencies of each category of verbal responses measured using the LIWC software was compared between groups using univariate ANOVAs.

In all analyses the threshold for significance was .05 two-tailed. Where group differences are reported, secondary analyses were performed to examine correlations between the dependent variable and scores on the hyperactivity/impulsivity and inattentiveness sub-scales of the CAARS in the ADHD group, computing Pearson’s r or Spearman’s rho depending on the data type. These analyses were performed to determine whether each aspect of impaired driving was explained by variability in hyperactivity-impulsivity or inattention symptoms or both.

Participants on atomoxetine (*n* = 2) were not withdrawn from medication for the study. To ensure this did not influence the findings, all analyses were re-run excluding these participants. The findings remained the same and the participants were not group outliers (defined as >2.5 SD from the group mean) on any measure. The results are therefore reported with these participants included.

The possible influence of comorbid ASD symptoms on the pattern of results was checked by re-running all ANOVAs for which there was a main effect of Group, with AQ scores included as a covariate, or by computing correlations between dependent variables and AQ scores. The analyses confirmed that AQ scores were not significantly related to any DV and did not alter the pattern of group effects reported in the results section. These secondary analyses are therefore not included in the Results section.

## Results

### Group characteristics

Of the fifty-nine participants enrolled in the study, 14 (6 ADHD and 8 control) were unable to complete the entire driving assessment due to simulator sickness. Data for 2 participants (1 ADHD and 1 control) were lost due to technical problems, leaving 22 participants in the ADHD group (16 males, age range 19–50 years, 14 on stimulant medication, 2 on atomoxetine) and 21 participants in the control group (18 males, age range 18–54) for analysis. For 2 participants no eye tracking data was available due to recording difficulties. The groups did not differ in age or gender distribution, and scores on the CAARS confirmed significant differences in ADHD symptoms between the groups (see Table 1). CAARS T-scores of those on prescribed medication (mean = 82.25, SD = 8.42) were similar to those not taking prescribed medication (mean = 83.17, SD = 8.11) although the numbers not taking medication were too small to permit formal statistical analysis.

**Table 1:** Group data for age, ADHD symptoms, self-reported driving behaviour and driving history

	Control (*N* = 21)	ADHD (*N* = 22)	Group difference
	Mean (SD)	Mean (SD)	t-value
Age	34.0 (13.8)	31.4 (10.2)	0.687
*CAARS (T score)*			
ADHD symptoms total	48.5(10.2)	82.5(8.5)	12.07**
Hyperactive-Impulsive symptoms	43.6(7.0)	73.6(9.3)	11.88**
Inattentive symptoms	50.7(15.3)	80.9(9.0)	7.82**
*Manchester Driving Behaviour Questionnaire*			
Errors	3.4(3.8)	4.7(4.2)	1.02
Violations	4.3(3.5)	11.2(7.0)	3.94**
Lapses	7.6(5.1)	15.1(7.0)	3.94**
*Driving history*			
Years since passing test	13.7(12.9)	10.6(8.7)	0.91
Times taken test	1.6(1.0)	2.1(1.9)	1.11
Number of accidents since test	0.95(0.92)	1.89(1.28)	2.65*
Accident serious enough for insurance claim	0.57(0.81)	1.55(1.88)	2.18*
Accidents involving injury	0.14(0.36)	0.32(0.48)	1.28
Penalty points on licence	0.14(0.65)	1.21(2.55)	1.77
Annual mileage	6186(5125)	10632(9135)	1.87
Number of hours driving a week	6.5(5.8)	8.5(8.9)	0.88

CAARS = Conners Adult ADHD Rating Scale. The data shown for the CAARS are group mean T-scores for which the standardised (population) mean score is 50 and the standard deviation is 10

*p < .05 **p < .01

### Self-report measures of driving behaviour and driving history

Group data for the DBQ and driving history are shown in Table 1. ADHD participants reported more accidents since passing their test than controls and this difference remained significant with annual mileage included as a covariate. ADHD participants reported significantly more Violations and Lapses than control participants but not Errors. Hyperactivity/impulsivity symptoms correlated significantly with Violations (r = .6, p < .001) and Lapses (r = .54, *p* < .001) and the rate of serious accidents (r = .32, *p* < .05). Inattentive symptoms correlated significantly with Violations (r = .52, *p* < .001) and Lapses (r = .58, *p* < .001).

### Simulator driving performance

A mixed design ANOVA (2 Groups × 2 Road Types) showed significant group differences in the proportion of distance travelled over the speed limit with the ADHD group speeding more than the control group (main effect Group) and more speeding on the motorway than in the urban area (main effect Road Type), but no Group by Road Type interaction (see Table 2). Average speed increased according to the speed limit for the particular road type, and was also higher for the ADHD group than control group, but there was no Group*Road Type interaction. As to be expected with frequent stopping and turning, the coefficient of variance of speed was highest in the urban area. There was no significant group difference or interaction. The standard deviation of the lateral position increased during the drive as expected with the increase in lane width and the opportunity to change lanes on the motorway, but again there was no significant group difference or Group × Road Type interaction. Average speed across road types correlated significantly with hyperactivity/impulsivity (r = .35, *p* < .05) and inattentive (r = .36, *p* < .05) scores on the CAARS.

**Table 2:** Group comparisons of simulator performance and eye movement measures for the urban and motorway routes

	Control		ADHD		Statistical analysis		
	Urban	Motorway	Urban	Motorway	Group	Road type	Group*Road type
Simulator variables					F(1,41)	F(1,41)	F(1,41)
Distance over speed limit^a^	0.08 (0.06)	0.16 (0.14)	0.14 (0,14)	0.25 (0.19)	4.40*	17.85**	0.37
Forward speed (mph)	14.9 (2.3)	62.4 (4.8)	16.7 (2.4)	66.0 (6.1)	5.96*	4522.67**	1.60
Coefficient variation velocity	0.70 (0.11)	0.15 (.05)	0.67 (0.12)	0.15 (0.06)	1.67	886.12**	1.17
Lane deviation^b^	0.55 (0.06)	0.99 (0.25)	0.53 (0.13)	0.94 (0.07)	1.22	193.17**	0.21
Eye movement variables					F(1,37)	F(1,37)	F(1,37)
Horizontal spread search	21.15 (14.79)	18.33 (13.13)	21.58 (14.97)	16.50 (14.09)	.06	27.79**	1.34
Vertical spread search	10.97 (7.55)	11.96 (7.16)	11.59 (9.65)	11.88 (10.90)	.07	5.81*	.85
Fixation duration (ms)	381 (89)	424 (150)	394 (130)	489 (200)	.16	5.22*	.06

*p < .05 ** p< .01

^a^Distance over speed limit is calculated as the proportion of the distance travelled in excess of 10 % of the speed limit for that road section plus two miles

^b^Lane deviation is calculated as the standard deviation of lateral position in metres

### Eye movement measures

Analysis of the eye movement measures revealed main effects of Road Type on fixation duration and horizontal spread of search, with shorter fixation durations and a larger horizontal spread of search in the urban area than on the motorway. Vertical spread of search was higher on the motorway than in the urban area. No group differences were found for fixation duration, horizontal spread or vertical spread were found and no interactions were observed on any measure.

There was no difference in reported total simulator sickness symptoms between the ADHD group and the control group either after the practice drive (*F* (1, 42) = 1.04, *p* = .31) or the test drive (*F* (1, 42) = .04, *p* = .85).

### Observational coding of driving behaviours

#### Driving performance

The analysis of the coded observations of driving behaviour showed poorer performance in ADHD than controls on a number of measures. To reduce the number of comparisons, multivariate analyses were conducted on categories of items, shown in Table 3 (univariate F-ratios are presented for information). Specifically ADHD participants showed poorer Vehicle Control, greater levels of Frustration/Anger and a trend for less safe driving when Changing Lanes/Overtaking on the motorway. There were no group differences in the Approach to Junctions, Use of Speed or Lane Positioning categories (all p > .1). CAARS Hyperactivity/Impulsivity scores correlated significantly with Vehicle Control (rho = .41, p < .01), Frustration/Anger (rho = .35, *p* < .05) and Changing Lanes/Overtaking on the Motorway (rho = .39, *p* = .01). CAARS Inattention scores correlated significantly with Vehicle Control (rho = .34, *p* < .05) but not Frustration/Anger or Changing Lanes/Overtaking on the Motorway (*p* > .05).

**Table 3:** Comparisons of the categories of observed driving behaviours in the ADHD and control groups

	Control (N = 21)	ADHD (N = 22)	Group comparison
	Mean observed frequencies (SD)		F-ratio
Approach to junctions (urban area)			**1.75**
No mirror check when turning	4.95(1.43)	4.00(2.16)	2.88*#*
No signal when turning	0.00(0.00)	0.05(0.22)	.95
Improperly timed signal	0.67(0.91)	0.71(0.90)	.01
Inadequate observations junction	0.00(0.00)	0.32(0.78)	3.49*#*
Changing lanes/overtaking (motorway)			**2.16** ***#***
Number of lane changes/overtakings	9.90(5.14)	13.20(6.83)	3.07*#*
No mirror check when changing lane	0.76(1.14)	2.24(3.03)	3.70*#*
No signal when changing lane	0.62(1.99)	1.57(2.50)	3.25*#*
Improperly timed signal changing lane	1.38(2.13)	2.14(3.57)	.01
Over speed limit when overtaking	0.05 (0.22)	0.23 (0.43))	3.48*#*
Use of speed			**1.48**
Not slowing with the vehicle ahead	0.00 (0.00)	0.09 (0.29)	2.00
Hesitancy	0.14 (0.36)	0.00 (0.00)	3.53#
Too slow for surrounding traffic	0.05 (0.22)	0.14 (0.47)	.62
Too fast approaching junctions	0.05 (0.22)	0.14 (0.47)	.62
Not slowing after speed sign	2.10 (1.29)	2.64 (1.09)	1.45
Lane positioning			**1.02**
Too close to the kerb	0.10 (0.30)	0.64 (1.50)	2.64
Too far from the kerb/out of lane	0.90 (1.14)	1.23 (1.48)	.64
Driving on the hard shoulder	0.14 (0.36)	0.27 (0.55)	.83
Vehicle control			4.28*
Hand off the wheel > 5 s	0.00 (0.00)	0.59 (1.18)	4.43*
Wrong gear	0.38 (0.59)	1.14 (1.64)	5.56*
Stalling	0.24 (0.54)	0.48 (0.98)	.95
Braking			**.63**
Braking too abruptly	0.05 (0.22)	0.14 (.047)	.63
Frustration/ Anger			4.65*
Unnecessarily using the horn	0.05 (0.22)	0.36 (1.00)	2.00
Impatience behind stationary/slow traffic	0.00 (0.00)	0.41 (0.67)	7.91**
Responses to events	Observed frequencies in each category		*Χ* ^2^ statistic
Urban			
Crashed (driver’s fault)/ near miss	0.10	0.18	**5.52 #**
Slowed down/ changed lane	0.74	0.55	
Drove past fast	0.16	0.26	
Motorway			
Did not slow down	0.29	0.25	**1.42**
Slowed but not below speed limit	0.11	0.19	
Slowed below speed limit	0.60	0.57	

The behaviours are grouped into categories, depicted in bold type. The between-subject multivariate analyses (conducted across items within each of the categories) are shown. Additional univariate F-ratios are presented for information for all categories except the Responses to Events

#### Responses to events

As shown in Table 3, there was a trend towards a significant group difference in the response to events in the urban route with more participants in the ADHD group causing a crash or near miss and driving past events too fast, and more participants in the control group stopping, slowing down or changing lanes. Additional analysis was performed to determine whether this pattern of effects may have been influenced by the mean speed in the period leading up to potential collision with the hazard (from the moment of hazard onset). Analysis confirmed that the ADHD group drove faster approaching the events than the control group, *F* (1, 40) = 8.44, *p* < .01. Responses to events on the motorway did not differ significantly between groups.

#### Emotional speech

ADHD participants made significantly more comments than controls and the comments made contained more words (see Table 4 for statistics). In particular they were more likely to express anger and swear at other road users. These dependent variables correlated significantly with CAARS hyperactivity/impulsivity scores (Anger: rho = .55, *p* < .001; Swearing: rho = .46, *p* < .01). Correlations with CAARS Inattentive scores were non-significant (p > .05).

**Table 4:** Group comparisons of frequencies of driving-related speech

	Control	ADHD	Group comparison
	Mean (SD)	Mean (SD)	F value
Number of comments	2.25 (4.74)	10.82 (15.98)	5.75*
Word count per comment	1.71 (2.09)	4.01 (3.25)	7.59**
Positive emotions	0.51 (0.91)	2.9 (6.38)	2.89
Negative emotions	0.75 (1.72)	6.03 (12.2)	3.86#
Anxiety	0.06 (0.23)	0.13 (0.45)	0.36
Anger	0.52 (1.37)	4.31 (6.78)	6.31*
Swearing	0.73 (2.14)	4.02 (6.46)	4.95*

#p < 0.1*p < .05 level **p < .01

## Discussion

As predicted and in line with previous research [3, 4, 6–9, 41], ADHD participants differed from healthy adults on a number of indicators of driving performance, specifically, average speed, proportion of the distance travelled over the speed limit, vehicle control (taking hands off the wheel, putting the car in the wrong gear), changing lanes/overtaking safely on the motorway (at a trend level of significance), reactions to sudden events and the expression of frustration or anger with other road users. The ADHD group also reported more driving offences and accidents, even after controlling for group differences in annual mileage and scored significantly higher on the Lapses and Violations sub-scales of the Manchester Driving Behaviour Questionnaire. In addition, speech analysis of in-car comments, revealed that the ADHD group made negative comments (anger and swearing) significantly more frequently than controls.

Contrary to our hypothesis, driving route did not influence ADHD performance (speed, speeding, variance of speed) or eye movements or fixation duration, differently from control subjects. Furthermore, ADHD participants were as likely as control participants to slow down on the motorway in response to traffic slowing ahead or a change in the speed limit. These results suggests that adults with ADHD can adjust and maintain speed appropriate to the driving environment and can orient attention and maintain focus on the road ahead when driving in a low stimulation driving environment for short periods of time (15 min). Previous studies have reported worse driving in ADHD subjects on a highway (motorway) than an urban route [22] and in the second of two low stimulation routes [24] but in both studies the simulator session lasted up to 60 min and so provided a greater challenge to sustained attention. Further research is needed to characterise more fully the time-course of effects of a low stimulus driving environment on driving performance in ADHD, but it is likely that the period of time used in the present study (approximately 30 min total drive) was not sufficient to detect these potentially subtle effects. It may also be important in future studies to consider the influence of the novelty of the driving simulator on ADHD symptoms as this may improve the regulation of arousal that is hypothesised to be disturbed in ADHD [15] and mitigate any effects of a low stimulation driving route. Indirect support for this interpretation comes from a recent meta-analysis which concluded that ADHD participants report driving more (greater annual mileage) than control participants and that this may be attributable to the stimulating effects of driving on attention [4].

Although able to respond well to events in the motorway section of the simulated drive, the ADHD group were less likely to change lanes and overtake safely on the motorway, often speeding when overtaking and failing to signal or use mirrors when changing lane. This is consistent with the greater average speed of the ADHD group across both the urban and motorway routes and the tendency to express frustration and anger with other road users, all of which indicate an impulsive driving style, consistent with evidence of impairment in behavioural and emotional self-regulation in ADHD [25, 26, 42]. Similarly, the ADHD participants’ responses to events in the urban area were less controlled than those of the control group in that they caused a near miss or crash more often, did not slow down and were less likely to alter the path of the vehicle (change lanes) when confronted with an event, possibly because they were travelling at greater speed than the control group when an event occurred and consequently had less time to react. When applied to real-world driving this suggests that impulsivity, and possibly weaker error detection and performance adjustment may be responsible for the increased rate of accidents in this population.

A number of studies have proposed inattention to be the main or most likely cause of accidents in healthy individuals [18] and those with ADHD [22, 24]. Our findings do not necessarily undermine the role of inattention in the poor driving and increased rates of accidents in ADHD, but they indicate a substantive role for the hyperactivity/impulsivity symptom dimension. In particular, the rate of serious accidents and the tendency towards verbally aggressive behaviour (swearing and angry comments) were predicted by hyperactivity/impulsivity symptoms. In line with a previous study of healthy adults with high ADHD traits [7] this suggests that difficulties regulating and controlling behaviours may lead to a tendency towards ‘road rage’ and may also lead to serious accidents. It is not clear at present why others [22, 24] have found a lesser role for hyperactive/impulsive symptoms in impaired driving in ADHD while we found this to be an important factor; further work is needed to determine whether differences between studies in the design of simulator routes (e.g. traffic level in the urban area, number of pre-determined events) and/or the provision of performance-based monetary incentives are responsible. It is also possible that differences in the degree of variability in scores on the two measures underlie the effects in the present study. In the present study, the distribution of scores was found to be comparable across variables.

Future studies should examine the impact of stimulant and non-stimulant medications on driving performance in ADHD and the degree to which any improvements following medication administration are related to reductions in hyperactivity/impulsivity and/or inattention symptoms. Although previous studies have reported improvements in a range of indices of driving performance following medication administration (e.g. [43–45], none have examined the extent to which these effects are driven by one or both symptom domains. Moreover, the potential for non-pharmacological interventions to enhance driving performance in ADHD has not yet been explored. These could include in-car driver monitoring, driver feedback (including biofeedback) and alerts and driver education, all of which may prove particularly useful where medication is not effective and/or adherence is poor.

Limitations of the present study include the sample size and composition. Previous studies have shown group differences in MBDQ errors as well as violations and lapses [9] suggesting that our study may have been under-powered to find an effect on errors. However, the effect is likely to be small in that case, and certainly less important than violations and lapses. In the current study there were also relatively few female participants, which may have affected the results as studies of healthy adults have shown that females report more errors than males [38]. Although more prevalent in males than females in childhood, there is an approximately equal gender ratio in community samples of adults with ADHD [46]. Future driving research should ensure that this equality is reflected in participant samples. Finally, in the present study, the oculomotor measures did not yield significant effects. This could reflect insufficient power to detect an effect, although one other study also reported no differences in oculomotor activity during driving in ADHD and controls [32]. Future research should employ other measures of attention and arousal regulation, such as heart rate variability, and should also determine whether contextual factors, such as the novelty of the driving simulator environment, normalise arousal in lab-based studies of driving in ADHD. In relation to this, the role of distractors such as mobile phones has received very little attention in previous research with one study showing equivalent levels of distraction among adolescents with and without ADHD [41]. Further work is needed to extend this to other distractors, such as car stereos and satellite navigation systems and to explore the cognitive factors that contribute to distraction during driving.

## Conclusions

This study examined driving performance in adults with ADHD during a simulated driving session. The ADHD group differed significantly from the typical adult control group on a number of measures, particularly those relating to the expression of frustration and anger during driving. Other differences included vehicle control and average speed as well as speed when overtaking on the motorway. The pattern of effects suggests that, in this sample, impaired driving may be related to problems with the control of emotions and motor actions, rather than inattention, although further research using other measures of attention is needed to determine whether this effect is reliable, and/or influenced by the simulator environment.
